# TIGIT inhibition and lenalidomide synergistically promote antimyeloma immune responses after stem cell transplantation in mice

**DOI:** 10.1172/JCI157907

**Published:** 2023-02-15

**Authors:** Simone A. Minnie, Olivia G. Waltner, Kathleen S. Ensbey, Stuart D. Olver, Alika D. Collinge, David P. Sester, Christine R. Schmidt, Samuel R.W. Legg, Shuichiro Takahashi, Nicole S. Nemychenkov, Tomoko Sekiguchi, Gregory Driessens, Ping Zhang, Motoko Koyama, Andrew Spencer, Leona A. Holmberg, Scott N. Furlan, Antiopi Varelias, Geoffrey R. Hill

**Affiliations:** 1 Clinical Research Division, Fred Hutchinson Cancer Center, Seattle, Washington, USA.; 2 QIMR Berghofer Medical Research Institute, Brisbane, Queensland, Australia.; 3 Translational Research Institute, Woolloongabba, Queensland, Australia.; 4 Hugh Green Cytometry Centre, Malaghan Institute of Medical Research, Wellington, New Zealand.; 5 iTeos Therapeutics, Gosselies, Belgium.; 6 Australian Center for Blood Diseases, Monash University and; 7 Malignant Haematology and Stem Cell Transplantation, The Alfred Hospital, Melbourne, Victoria, Australia.; 8 Department of Clinical Haematology, Monash University, Melbourne, Victoria, Australia.; 9 Division of Medical Oncology and; 10 Department of Pediatrics, University of Washington, Seattle, Washington, USA.; 11 Faculty of Medicine, The University of Queensland, St. Lucia, Queensland, Australia.

**Keywords:** Immunology, Oncology, Adaptive immunity, Bone marrow transplantation, Cancer immunotherapy

## Abstract

Autologous stem cell transplantation (ASCT) with subsequent lenalidomide maintenance is standard consolidation therapy for multiple myeloma, and a subset of patients achieve durable progression-free survival that is suggestive of long-term immune control. Nonetheless, most patients ultimately relapse, suggesting immune escape. TIGIT appears to be a potent inhibitor of myeloma-specific immunity and represents a promising new checkpoint target. Here we demonstrate high expression of TIGIT on activated CD8^+^ T cells in mobilized peripheral blood stem cell grafts from patients with myeloma. To guide clinical application of TIGIT inhibition, we evaluated identical anti-TIGIT antibodies that do or do not engage FcγR and demonstrated that anti-TIGIT activity is dependent on FcγR binding. We subsequently used CRBN mice to investigate the efficacy of anti-TIGIT in combination with lenalidomide maintenance after transplantation. Notably, the combination of anti-TIGIT with lenalidomide provided synergistic, CD8^+^ T cell–dependent, antimyeloma efficacy. Analysis of bone marrow (BM) CD8^+^ T cells demonstrated that combination therapy suppressed T cell exhaustion, enhanced effector function, and expanded central memory subsets. Importantly, these immune phenotypes were specific to the BM tumor microenvironment. Collectively, these data provide a logical rationale for combining TIGIT inhibition with immunomodulatory drugs to prevent myeloma progression after ASCT.

## Graphical abstract

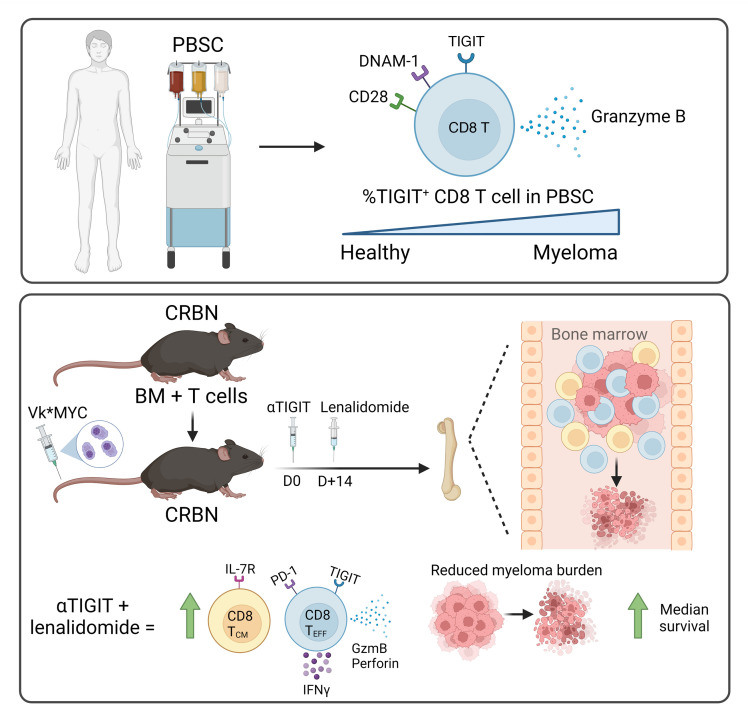

## Introduction

Autologous stem cell transplantation (ASCT) is an effective and highly utilized consolidative therapy for multiple myeloma (MM) that prolongs progression-free survival (PFS). We have previously shown that T cell–dependent myeloma-specific immunity contributes to prolongation of PFS and that disease progression is characterized by immune escape (1, 2). This immune escape is associated with T cell dysfunction and the expression of inhibitory receptors, such as TIGIT, in both patients and preclinical models (2, 3). Consistent with this, inhibition of TIGIT reduced myeloma progression in transplant and nontransplant experimental systems (2, 3). Several clinical studies have shown expression of multiple inhibitory receptors on CD8^+^ T cells in peripheral blood (PB) of patients with MM (4–6). In vitro data suggested that PD-1 inhibition may provide additive effects with immunomodulatory drugs (IMiDs) (7, 8). However, in phase III clinical trials (KEYNOTE-183 and KEYNOTE-185) of the combination of anti–PD-1 (αPD-1) antibodies and IMiDs (9, 10), there was an increase in serious adverse events in the αPD-1 arms and both trials were stopped early due to an unfavorable risk-benefit profile and decreased overall survival. The significantly increased expression of TIGIT relative to other immune checkpoints, including PD-1, in patients with MM suggests TIGIT may be a favorable target (3). In the present study, we provide preclinical evidence to support the combination of TIGIT inhibition with IMiDs as maintenance therapy after SCT. Moreover, we demonstrate that the antitumor activity of αTIGIT in MM is dependent on Fc–Fcγ receptor (Fc-FcγR) coengagement.

## Results

### TIGIT is upregulated on nonsenescent CD8^+^ T cells in mobilized PB stem cell products.

**Figure 1:**
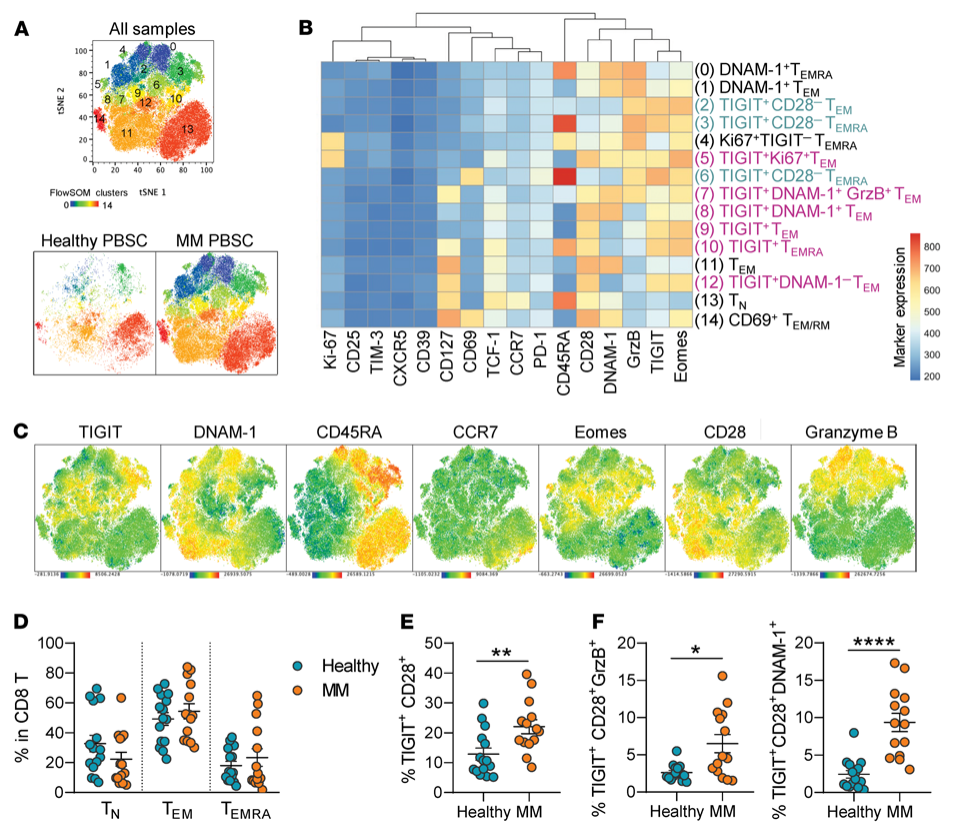
TIGIT expression is upregulated on CD8^+^ T cells in peripheral blood stem cell grafts from patients with multiple myeloma undergoing ASCT. (**A**–**F**) Mobilized peripheral blood stem cell (PBSC) satellite vials from patients undergoing ASCT for multiple myeloma (MM) were thawed and stained for analysis via flow cytometry alongside PBSCs from healthy patients (*n* = 14 for MM PBSCs; *n* = 15 for healthy PBSCs). (**A**) t-Distributed stochastic neighbor embedding (t-SNE) plots of CD8^+^ T cells, colored by FlowSOM populations, in MM PBSCs (*n* = 14) and healthy PBSCs (*n* = 3). (**B**) Heatmap of marker expression (MFI) across FlowSOM CD8^+^ T cell populations. TIGIT^+^ populations are colored blue (CD28^–^) or purple (CD28^+^) to indicate putative senescence versus activation, respectively. (**C**) t-SNE plots from all samples in **A** colored by expression of markers of interest. FlowSOM heatmap, and marker expression t-SNE plots for the remaining healthy PBSC samples (*n* = 12) are presented in Supplemental Figure 1. (**D**) Frequency of naive (Tn; CCR7^+^CD45RA^+^), effector memory (Tem; CCR7^–^CD45RA^–^), and terminally differentiated (Temra; CCR7^–^CD45RA^+^) subsets within CD8^+^ T cells. (**E**) Frequency of all TIGIT^+^CD28^+^ cells within CD8^+^ T cells. (**F**) Frequency of TIGIT^+^CD28^+^ subsets with coexpression of granzyme B (GrzB^+^) or DNAM-1 within CD8^+^ T cells. Data represent mean ± SEM. *P < 0.05, **P < 0.01, ****P < 0.0001 by Mann-Whitney test.

We sought to interrogate TIGIT expression in ASCT donor products, as clinical studies have reported expression of TIGIT on bone marrow (BM) CD8^+^ T cells from patients with MM (3, 11) and we have previously shown that preexisting CD8^+^ T cells in the donor graft provide antimyeloma immunity in preclinical murine models (1). Thus, we performed high-parameter flow cytometry on CD8^+^ T cells from mobilized PB stem cell (PBSC) grafts to determine whether TIGIT is expressed on T cells that are putatively expanded during ASCT. We compared the phenotype of CD8^+^ T cells in PBSC grafts from patients with MM undergoing ASCT to PBSC grafts mobilized from healthy patients. The median age of patients at the time of mobilization was 62 ± 10 years in MM patients and 56 ± 16 years in healthy controls. We analyzed flow cytometry data using FlowSOM (12), a dimensionality reduction tool, to unbiasedly identify CD8^+^ T cell populations based on expression of various T cell markers. FlowSOM analysis of CD8^+^ T cells from both cohorts identified 15 populations spanning T cell differentiation, including naive (Tn; CCR7^+^CD45RA^+^), effector memory (Tem; CCR7^–^CD45RA^–^), and terminally differentiated (Temra; CCR7^–^CD45RA^+^) T cells (Figure 1, A–C, and Supplemental Figure 1, A–C; supplemental material available online with this article; https://doi.org/10.1172/JCI157907DS1). Quantities of naive and memory T cells were comparable between PBSCs from patients with MM compared with healthy controls (Figure 1D). Coexpression of costimulatory molecules (DNAM-1, CD28), inhibitory receptors (TIGIT, PD-1), transcription factors (TCF1, Eomes), and functional markers (Ki67, granzyme B) identified several subsets of Tem and Temra cells that expressed TIGIT (Figure 1, B and C). Interestingly, PD-1 was only dimly expressed on 1 subset of putatively senescent Temra cells (Figure 1B). Due to the age of the patient cohorts and the correlation of TIGIT expression with immune senescence, we divided TIGIT-expressing populations into those that were activated or senescent based on gain or loss of CD28 expression, respectively (Figure 1B) (13, 14). PBSC grafts from patients with MM had higher frequencies of activated TIGIT^+^CD28^+^CD8^+^ T cells compared with healthy controls (Figure 1E). We next evaluated coexpression of DNAM-1 on TIGIT-expressing cells, as expression of DNAM-1 on CD8^+^ T cells is necessary for the antitumor efficacy of TIGIT inhibition (15–17). Importantly, the frequency of TIGIT^+^CD28^+^ T cells that coexpressed DNAM-1 or granzyme B was significantly increased in patients with MM and these subsets thus likely represent the optimal targets for TIGIT inhibition (Figure 1F).

Next, to determine whether the stem cell mobilization process itself impacted T cell phenotype, we compared CD8^+^ T cells from PB mononuclear cells (PBMCs), both before and after G-CSF administration, to CD8^+^ T cells in the apheresis product from healthy controls. CD8^+^ T cells in PBMCs after G-CSF were more naive, with a reduced frequency of Tem and TIGIT^+^CD28^+^ T cells (Supplemental Figure 2, A and B). However, there was no difference in naive and memory T cell subsets in the apheresis product compared to pre–G-CSF PBMC samples, indicating that the increase in frequency of circulating naive T cells after G-CSF mobilization did not impact T cell composition in the apheresis product. On the other hand, expression of DNAM-1 on TIGIT^+^CD28^+^ T cells was marginally reduced after exposure to G-CSF in both PBMCs and in the apheresis product (Supplemental Figure 2C). The addition of plerixafor to G-CSF mobilization in patients with MM did not impact the frequency of TIGIT^+^CD28^+^ T cells in PBSC grafts (Supplemental Figure 2D). Furthermore, 2 patients were consecutively mobilized with G-CSF alone and then with G-CSF and plerixafor, which allowed for paired analyses of T cell phenotypes, and no differences in T cell subsets or the frequency of TIGIT^+^CD28^+^ T cells was observed (Supplemental Figure 2, E and F). Finally, we assessed the effects of cryopreservation on T cell differentiation and the frequency of TIGIT^+^CD28^+^ T cells in healthy PBMCs from young individuals. We observed a decrease in Tn cells with a subsequent increase in Temra; however, TIGIT^+^CD28^+^ T cells were unaffected by the cryopreservation process (Supplemental Figure 2, G and H). Together, these data highlight that activated TIGIT^+^DNAM-1^+^ T cells are present in G-CSF–mobilized PBSC grafts from patients with MM and as such TIGIT represents a logical immunotherapeutic target in combination with ASCT.

### Antimyeloma activity of αTIGIT antibodies after SCT is dependent on FcγR binding.

**Figure 2:**
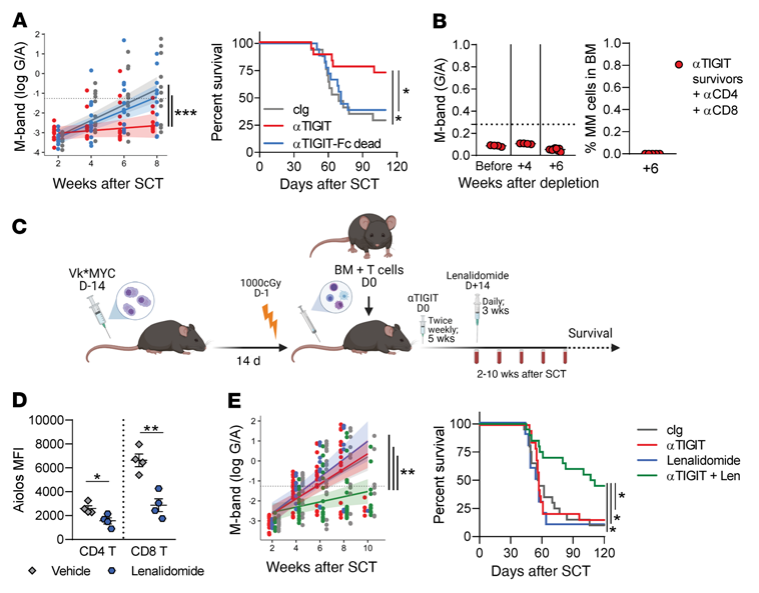
TIGIT blockade in combination with lenalidomide provides synergistic antimyeloma responses after SCT. (**A**) M-band and median survival of Vk12653-bearing C57BL/6J (B6) recipients transplanted with 10 × 10^6^ BM with 5 × 10^6^ T cells from B6 donors and then treated with 100 μg of Fc-live anti-TIGIT (αTIGIT; mouse surrogate for EOS-448), Fc-dead anti-TIGIT (αTIGIT-Fc dead), or mIgG2a isotype control (cIg) twice a week from day 0 to 5 weeks after SCT (*n* = 17–18/group; 3 experiments). Recipients were monitored for survival and M-band (log[gamma/albumin]) in the serum. M-bands were analyzed using mixed-effects longitudinal modeling with shaded confidence intervals and a predicted rate of tumor growth (solid line). Dotted line in all M-band graphs represents statistically determined relapse threshold. (**B**) M-band, and the frequency of myeloma cells in BM at 6 weeks after T cell depletion, from αTIGIT-treated long-term survivors (>100 days after SCT) that were administered depleting antibodies against CD4 (250 μg) and CD8 (150 μg) to eliminate T cell–mediated immunity (*n* = 4, 1 experiment; week 6 M-band *n* = 9, 2 experiments). (**C**–**E**) B6 or CRBN recipients were transplanted with 10 × 10^6^ BM with 2 × 10^6^ T cells from B6 or CRBN donors and then treated with 100 μg of αTIGIT or cIg twice a week from day 0 and daily lenalidomide (50 mg/kg) or vehicle from day +14 until 5 weeks after SCT. (**C**) Experimental design. (**D**) MFI of Aiolos in CD4^+^ and CD8^+^ T cells from blood of CRBN mice treated with lenalidomide or vehicle (*n* = 4/group). (**E**) M-band modeled as described and median survival (*n* = 10–20/group; 2 experiments). Median survival analyzed with log-rank test and Benjamini-Hochberg correction for multiple comparisons. Tukey’s test was performed to correct for multiple comparisons for modeled M-bands. Student’s 2-tailed *t* test was used for comparison of 2 data sets. Data represent mean ± SEM. *P < 0.05, **P < 0.01, ***P < 0.001.

Human αTIGIT antibodies under clinical investigation either bind FcγR (αTIGIT) or are modified to limit Fc binding (αTIGIT-Fc-dead) (18). Elucidating the contribution of FcγR-binding to αTIGIT activity is crucial for the clinical development of these agents in hematological cancers, including MM, as mouse models have demonstrated necessity for FcγR binding in solid tumors (19). Thus, we determined the contribution of FcγR binding to antimyeloma activity of a murine αTIGIT antibody. We used our syngeneic SCT systems characterized by CD8^+^ T cell control since the potency of immune control in our ASCT model, where donor T cells are myeloma experienced, makes any substantial increases in survival with checkpoint inhibition difficult to ascertain (1). We administered αTIGIT or αTIGIT-Fc-dead early after SCT and found that immunological efficacy of TIGIT inhibition was highly dependent on FcγR binding (Figure 2A). Furthermore, mice that responded to αTIGIT treatment were cured of myeloma, as depletion of T cells in long-term survivors did not result in subsequent disease relapse (Figure 2B). Nonetheless, a subset of mice did not respond to αTIGIT treatment and may benefit from combinational approaches.

### The combination of αTIGIT and lenalidomide promoted synergistic antimyeloma activity.

IMiDs are standard of care as maintenance therapy after SCT and both stimulate immunity and directly inhibit myeloma cells. We thus hypothesized that TIGIT blockade may provide synergistic antimyeloma activity when combined with IMiDs after ASCT. MM-bearing mice were transplanted with a suboptimal dose of T cells followed by treatment with αTIGIT and/or lenalidomide (Figure 2C). We confirmed reduced expression of the IMiD target Aiolos in T cells from lenalidomide-treated CRBN mice in our model (Figure 2D). There was no monotherapeutic activity of αTIGIT or lenalidomide with this suboptimal T cell dosing; however, the combination of both drugs provided synergistic antimyeloma efficacy (Figure 2E). We observed no increase in toxicity (clinical score [ref. 20] and weight loss) in combination-treated mice compared to either monotherapy (data not shown). These data confirm that the combination of an IMiD with αTIGIT is an effective approach to target monotherapy nonresponders.

### αTIGIT and lenalidomide combination immunotherapy expands polyfunctional effector CD8^+^ T cells early after transplantation.

**Figure 3:**
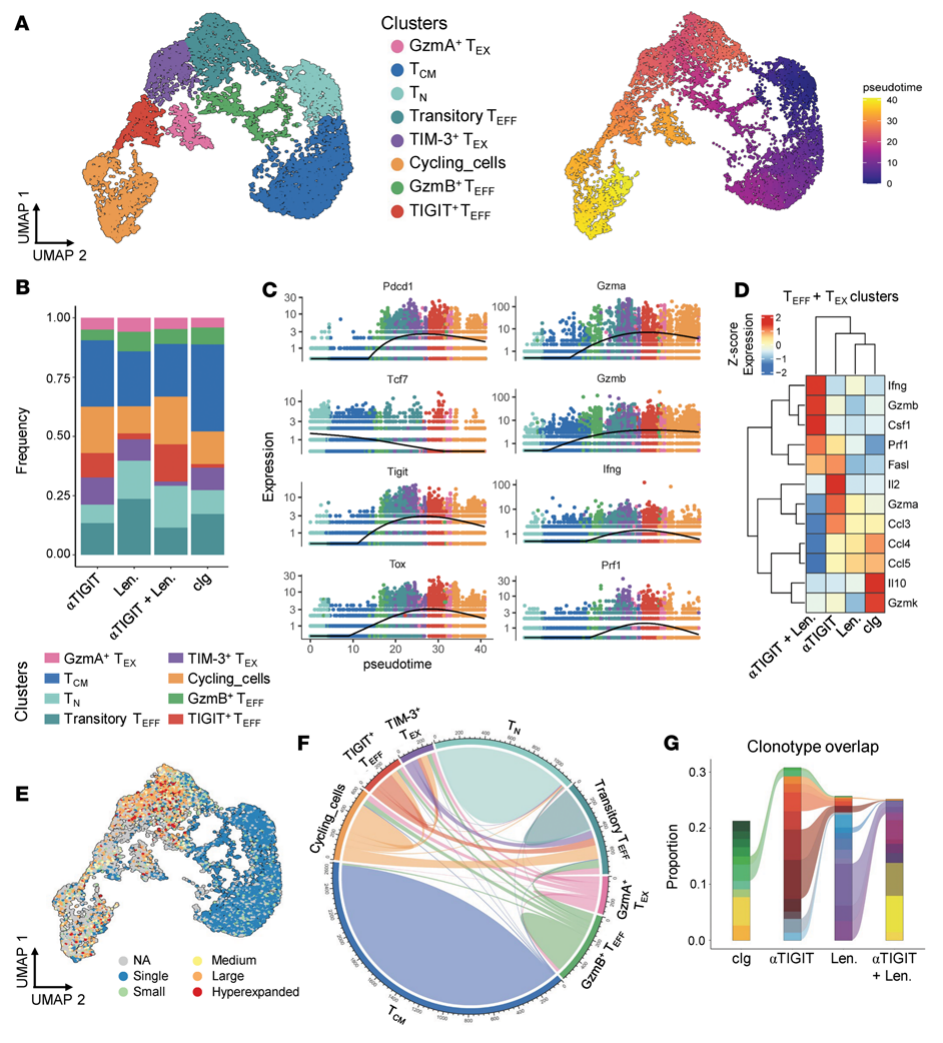
The combination of αTIGIT and lenalidomide expands polyfunctional effector CD8^+^ T cells early after transplantation. CRBN or B6 recipients were transplanted with 10 × 10^6^ BM with 2 × 10^6^ T cells from CRBN or B6 donors and then treated with 100 μg of αTIGIT or isotype control (cIg) twice a week from day 0 and daily lenalidomide (50 mg/kg; Len.) or vehicle from day +14 until 4 weeks after SCT. Mice were sacrificed on week 4 and CD8^+^ T cells were sorted for 5′ single-cell RNA sequencing (*n* = 5/group). (**A**) UMAP of BM CD8^+^ T cells colored by cluster (left) and pseudotime trajectory analysis (right). (**B**) Frequency of each cluster across treatment groups. (**C**) Expression of genes of interest over pseudotime colored by clusters. (**D**) Heatmap of expression of functional genes in Teff and Tex clusters across treatment groups. (**E**) UMAP colored by TCR clone size: small, >1 and ≤5; medium, >5 and ≤20; large, >20 and ≤100; hyperexpanded, >100 and ≤500. (**F**) Clonotype overlap between clusters. (**G**) Clonotype overlap between treatment groups. No overlap is observed between cIg and αTIGIT + Len.

We have previously demonstrated that CD8^+^ T cells are the major mediators of myeloma-specific T cell responses and that TIGIT inhibition activates CD8^+^ T cells after SCT (1, 2). Thus, we performed single-cell RNA sequencing to measure changes in both gene expression and the TCR repertoire in CD8^+^ T cells from the BM of mice treated with either murine IgG2a isotype control (cIg), monotherapies, or the combination of αTIGIT and lenalidomide. Analysis was performed 4 weeks after SCT to limit the impact of tumor burden on CD8^+^ T cell phenotypes. Unbiased clustering identified 8 CD8^+^ T cell clusters, including several effector (Teff) and exhausted (Tex) T cell subsets (Figure 3A). A population of *Tigit-*expressing Teff cells expanded in response to TIGIT inhibition, particularly in combination-treated mice (Figure 3B). Combination-treated mice also had a reduced frequency of TIM-3^+^ Tex cells relative to all other treatment groups (Figure 3B). Across the pseudotime trajectory from Tn, through transitory effector cells (21), to Teff and Tex subsets (Figure 3A), we observed an increase in the expression of inhibitory receptors, *Pdcd1* (PD-1) and *Tigit* (TIGIT), and a decrease in the stemness marker *Tcf7* with a subsequent increase in *Tox* expression (Figure 3C). As expected, expression of genes encoding functional molecules (granzymes, IFN-γ, and perforin) also increased over pseudotime in Teff and Tex subsets prior to diminished expression in cycling cells at the end of the trajectory (Figure 3C). However, within Teff and Tex clusters, we found significantly higher expression of *Ifng, Gzmb*, and *Prf1* in combination-treated mice (Figure 3D). Together, these data indicate that not only were Teff cells expanded in combination-treated mice, but the functional capacity of these cells was enhanced by combination immunotherapy early after transplantation.

We next assessed the clonality of T cells in our data set across clusters as a measure of putative tumor specificity. Overlap of T cell clones throughout differentiation stages suggests an ongoing T cell response to antigen. Indeed, we observed increased clonality measured by Simpson’s Clonality Index (Supplemental Figure 3A), with an abundance of hyperexpanded T cell clones (detected >100 copies), within Teff and Tex clusters (Figure 3E). Furthermore, there was substantial clonal overlap across clusters, particularly between transitory Teff and TIM-3^+^ Tex and TIGIT^+^ Teff populations (Figure 3F), consistent with previous literature describing transitory Teff cells as intermediaries to terminal differentiation in cancer and chronic infection (21, 22). Finally, we observed shared clonotypes across treatment groups, with several clones found in all immunotherapy-treated groups but not in the cIg-treated group (Figure 3G and Supplemental Figure 3B). In sum, immunotherapy clonally expanded shared TCRs after SCT that were largely absent in cIg-treated mice and that were found across CD8^+^ T cell differentiation stages. Together, these data suggest that combination immunotherapy likely drives the expansion of highly functional, myeloma-specific, effector CD8^+^ T cells after SCT. Nevertheless, myeloma specificity cannot definitively be proven without the use of tetramers and known myeloma antigens, which is acknowledged as a current limitation of the Vk*MYC myeloma model studied here.

**Figure 4:**
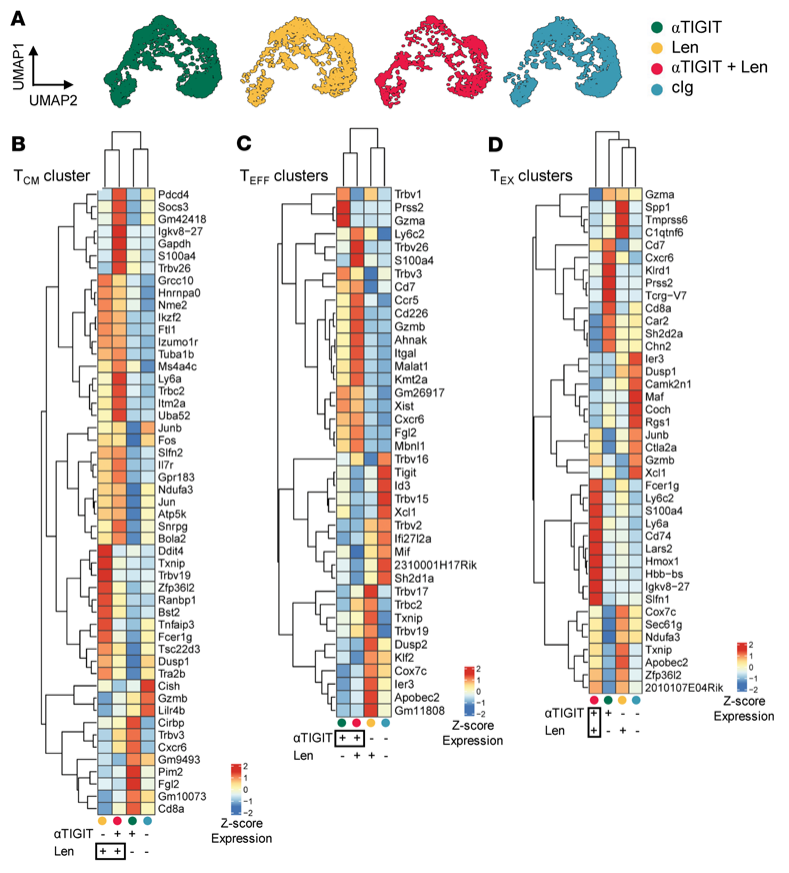
Gene expression in CD8^+^ T cell subsets is differentially driven by αTIGIT and lenalidomide. CRBN or B6 recipients were transplanted with 10 × 10^6^ BM with 2 × 10^6^ T cells from CRBN or B6 donors and then treated with 100 μg of αTIGIT or isotype control (cIg) twice a week from day 0 and daily lenalidomide (50 mg/kg; Len.) or vehicle from day +14 until 4 weeks after SCT. Mice were sacrificed on week 4 and CD8^+^ T cells were sorted for 5′ single-cell RNA sequencing (*n* = 5/group). (**A**) UMAPs of BM CD8^+^ T cells split by treatment group. Heatmaps with hierarchical clustering of top differentially expressed genes in (**B**) the central memory T cell cluster (Tcm), (**C**) effector T cell clusters (Teff), and (**D**) exhausted T cell clusters (Tex) across treatment groups.

To potentially determine the relative contributions of each monotherapy to the synergistic tumor control observed in combination-treated mice, we generated hierarchically clustered heatmaps depicting top differentially expressed genes across the 3 major CD8^+^ T cell phenotypes. Interestingly, even though all clusters were found across treatment groups (Figure 4A), albeit at different frequencies (Figure 3B), the gene expression profile of cells within each cluster was significantly altered by each monotherapy (Figure 4, B–D). Clusters were grouped into 3 main differentiation stages (central memory T cell [Tcm], Teff, and Tex) and treatment groups were hierarchically clustered based on differentially expressed genes. In the Tcm cluster, mice that received lenalidomide either alone or in combination with αTIGIT clustered together (Figure 4B), suggesting that lenalidomide was the main driver of Tcm phenotypes. Expression of *Ikzf2* (Ikaros) was increased in lenalidomide-treated mice along with *Il7r*, which is associated with maintenance and promotion of memory T cells (23, 24). In the Teff populations, clustering was driven by exposure to αTIGIT, which increased expression of genes encoding granzyme B (*Gzmb*), DNAM-1 (*Cd266*), and CXCR6 (*Cxcr6*). In Tex cells, combination-treated mice clustered independently of other treatment groups and exhibited increased expression of genes associated with a self-reactive innate-like T cell subset (i.e., *Fcer1g*) (25). These data suggest that memory T cell retention is mediated by lenalidomide, while effector T cell function is enhanced by αTIGIT. Moreover, the combination of lenalidomide and αTIGIT additionally modifies exhausted T cells.

### αTIGIT and lenalidomide limit CD8^+^ T cell exhaustion and promote accumulation of Tcm cells in the BM.

**Figure 5:**
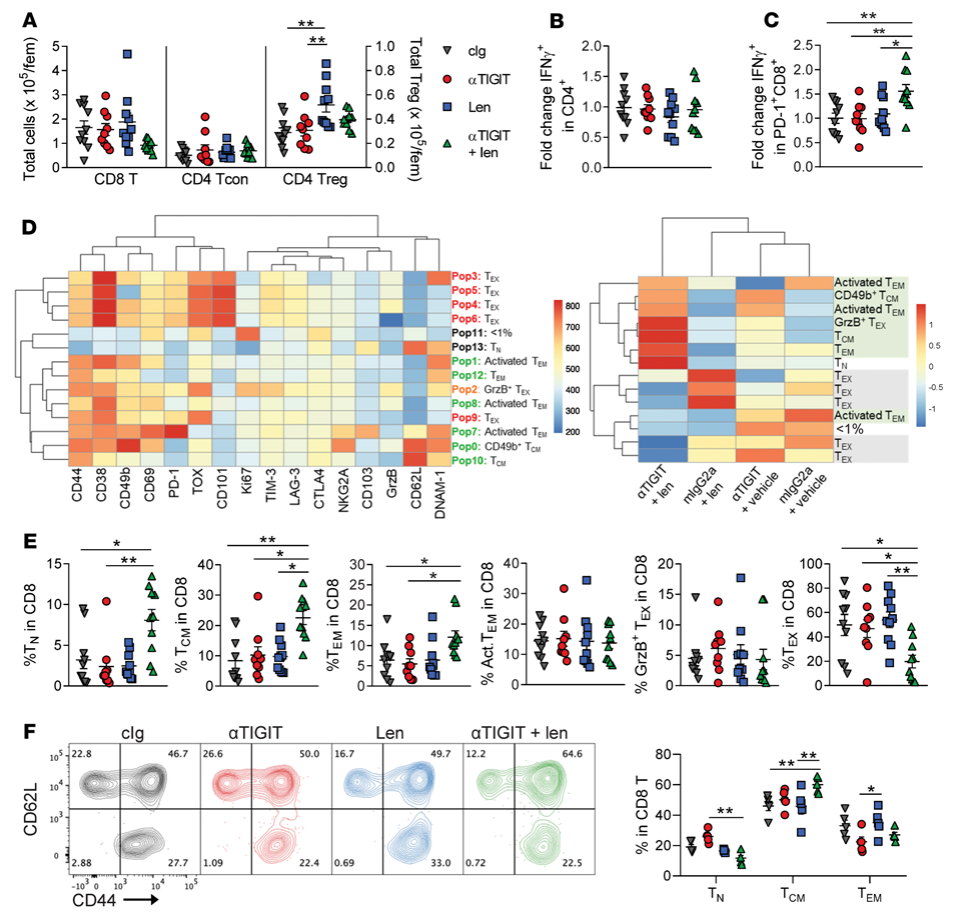
The combination of αTIGIT and lenalidomide reduces CD8^+^ T cell exhaustion and expands central memory T cells in the BM. CRBN or B6 recipients were transplanted with 10 × 10^6^ BM with 2 × 10^6^ T cells from CRBN or B6 donors and then treated with 100 μg of αTIGIT or cIg twice a week from day 0 and daily lenalidomide (50 mg/kg) or vehicle from day +14 until 5 weeks after SCT. Mice were sacrificed on week 6 and BM was harvested for analysis by flow cytometry (BM: *n*=10/group from 2 independent experiments). (**A**) Enumeration of T cell subsets in BM per femur (fem). CD8^+^ T and CD4^+^ conventional T (Tcon) cells on left axis and Tregs on right axis. (**B** and **C**) BM was stimulated with PMA/ionomycin to measure IFN-γ production as a fold change relative to the mean of cIg-treated mice in (**B**) CD4^+^ and (**C**) CD8^+^ Tcon cells. (**D**) Representative heatmap of marker expression (MFI) in each population of CD8^+^ T cells identified using FlowSOM (left) and heatmap of the relative mean frequency of each population across treatment groups (right). Tn = CD62L^+^CD44^–^, Tcm = CD62L^+^CD44^+^, Tem = CD62L^–^CD44^+^, Tex = TOX^+^. (**E**) Quantification of broader phenotypes (including one or more populations identified by FlowSOM) across treatment groups. Descriptions of individual populations and how they are grouped is included in Supplemental Table 1. (**F**) Recipients were transplanted with 10 × 10^6^ BM from naive donors with 2 × 10^6^ T cells from concurrent donor cohorts injected with myeloma at the same time as the recipients. Recipients were treated as above after SCT. Representative flow cytometry plots of CD62L and CD44 expression on CD8^+^ T cells from BM 4 weeks after SCT and quantification of CD8^+^ T cell differentiation. One-way ANOVA with Holm-Sidak’s test or Kruskal-Wallis test with Dunn’s multiple-comparison test. Data represent mean ± SEM. *P < 0.05, **P < 0.01.

To confirm the RNA sequencing data at the protein level, we interrogated CD8^+^ T cell phenotypes at a later time point after SCT when memory T cell accumulation would have occurred after contraction of Teff cell phenotypes in mice with controlled myeloma. High-parameter flow cytometry analyses of BM and PB T cells was undertaken 6 weeks after SCT. We observed modest effects on CD4^+^ T cells, namely a lack of lenalidomide-induced Treg expansion in combination-treated mice (Figure 5, A and B, and Supplemental Figure 4). The combination therapy increased IFN-γ production from PD-1^+^CD8^+^ T cells (Figure 5C), indicating that the enhanced functionality observed 4 weeks after SCT was maintained at this later time point. Most strikingly, the combination approach decreased the frequency of exhausted CD8^+^ T cells (Tex) and increased the frequency of Tcm subsets compared with monotherapy- and cIg-treated mice (Figure 5, D and E, and Supplemental Figure 5). We have previously demonstrated a strong correlation between the increased frequency of Tex and myeloma burden in the BM of mice that have relapsed after SCT, which subsequently results in reduced frequencies of memory T cells (2). To address this, we used a second SCT model, with myeloma-experienced T cells from myeloma-bearing donors (1), where myeloma was controlled in all groups at the time of analysis. Notably, the combination of αTIGIT and lenalidomide still promoted an increase in CD8^+^ Tcm cells above that of cIg-treated mice in this model (Figure 5F). These data demonstrate that the enhancement of memory T cells in combination-treated mice occurs independently of myeloma burden. Importantly, we have previously shown that memory CD8^+^ T cells are key mediators of myeloma immunity after ASCT (1).

Finally, phenotypic changes were BM specific, as we observed no effects on CD8^+^ T cells in PB (Supplemental Figure 6, A–C). This highlights that immunological mechanisms of action will likely be overlooked if BM samples are not analyzed alongside PB in clinical studies. We also observed no effect of the combination therapy on NK cell numbers compared to monotherapy-treated mice (Supplemental Figure 7A), and our preclinical transplantation model has previously not demonstrated a role for NK cells in post-SCT myeloma immunity (1). Lastly, the myeloid cell compartment in the BM was not altered by monotherapies or the combination approach (Supplemental Figure 7, A–C). Together, these data indicate that T cells in the BM were likely the key mediators of antitumor efficacy in mice treated with the combination of αTIGIT and lenalidomide.

### The antimyeloma efficacy of αTIGIT and lenalidomide is highly dependent on CD8^+^ T cells in the donor graft.

**Figure 6:**
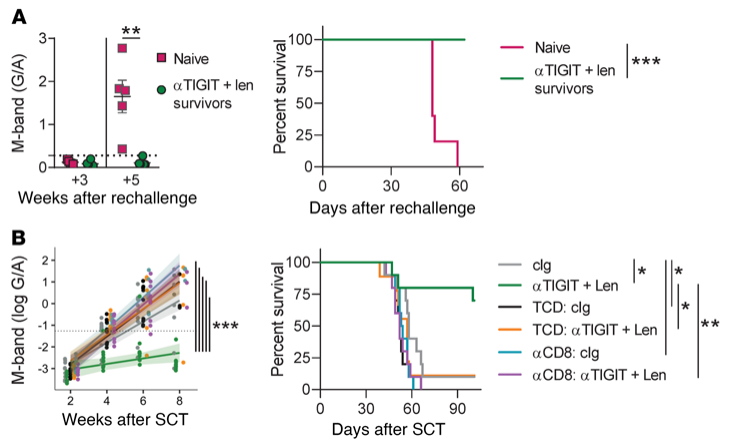
αTIGIT and lenalidomide generate immunological memory that is highly dependent on CD8^+^ T cells in the donor graft. (**A** and **B**) B6 or CRBN recipients were transplanted with 10 × 10^6^ BM with 2 × 10^6^ T cells from B6 or CRBN donors and then treated with 100 μg of αTIGIT or cIg twice a week from day 0 and daily lenalidomide (50 mg/kg) or vehicle from day +14 until 5 weeks after SCT. (**A**) M-band in long-term survivors after αTIGIT + lenalidomide treatment that were rechallenged with Vk12653 compared to naive controls (*n* = 5–6/group). G/A, gamma/albumin ratio. (**B**) Modeled M-band and overall survival of recipients transplanted as above or with T cell–depleted BM (TCD) or treated with CD8-depleting antibodies (αCD8). Median survival analyzed with log-rank test and Benjamini-Hochberg correction for multiple comparisons. Tukey’s test was performed to correct for multiple comparisons for modeled M-bands. Mann-Whitney was used for comparison of 2 data sets. Data represent mean ± SEM. *P < 0.05, **P < 0.01, ***P < 0.001.

To confirm that the immunological memory generated by the combination of αTIGIT and lenalidomide was functional, we rechallenged mice that had survived long term after combination treatment and found that all mice were protected against myeloma compared with naive controls (Figure 6A). Next, we sought to determine whether antimyeloma activity in combination-treated mice was mediated by T cells in the donor graft versus recipient cells that survived irradiation and donor T cells reconstituting from the BM. To achieve this, MM-bearing recipient mice were transplanted with BM and T cell grafts or with T cell–depleted (TCD) BM and treated with either αTIGIT and lenalidomide or cIg and vehicle (Figure 6B). The combination approach had no effect on myeloma growth in the absence of mature donor T cells within the graft, as no difference in M-band or median survival was observed between TCD recipients treated with αTIGIT and lenalidomide versus controls. Furthermore, depletion of CD8^+^ T cells completely abrogated the antimyeloma efficacy of the combination therapy in the recipients of T cell–replete grafts. In sum, these data highlight that the combination of αTIGIT and lenalidomide generated myeloma-specific immunity after SCT that was dependent on donor CD8^+^ T cells.

## Discussion

MM remains largely incurable despite advances in immunotherapies and the development of novel drugs. Here we discovered that the combination of an immunotherapy, αTIGIT, with a standard-of-care immunomodulatory drug, lenalidomide, generated synergistic antimyeloma effects. To date, immunotherapies targeting immune checkpoints in MM, such as PD-1, have had limited clinical efficacy and the combination of αPD-1 with IMiDs in phase III clinical trials (KEYNOTE-183 and KEYNOTE-185) was associated with decreased overall survival (3, 9, 10, 26). Response to immune checkpoint inhibition (ICI) requires expression of the targeted inhibitory receptors (IRs) on the right cells, in the right location. In MM, we and others have now demonstrated that TIGIT is the most upregulated IR on CD8^+^ T cells in both the BM and, shown here, autologous PBSC grafts (3). Furthermore, our data highlight that TIGIT expression was specifically upregulated on activated CD8^+^ T cells in patient PBSCs, which coexpressed DNAM-1 and/or granzyme B. Notably, myeloma cells from patients, and the Vk*MYC myeloma cells used in our preclinical models, express the ligands for PD-1 and TIGIT, PD-L1, and CD155, respectively (1, 3, 27–29). The magnitude of PD-1 versus TIGIT upregulation in patients with MM, both in the BM and here in PBSC samples, may provide some insight into the disappointing clinical activity of αPD-1 in MM. Nevertheless, it is important to quantify TIGIT expression in the context of costimulatory markers in older patients, particularly CD28 and DNAM-1, as TIGIT upregulation on CD8^+^ T cells is also associated with immunosenescence and aging (14, 30, 31). Recent data also highlight the necessity of DNAM-1 expression on CD8^+^ T cells to mount an effective antitumor response to TIGIT inhibition (15, 32). We and others have shown that αTIGIT efficacy is dependent on FcγR activity in solid tumor models (19, 33). Studies performed in vitro with human cells and in vivo preclinical models suggest that FcγR binding induces activation of NK cells and antigen-presenting cells and depletes TIGIT-expressing Treg cells and tumor cells through direct antibody-dependent cell-mediated cytotoxicity (19, 33). Here, we demonstrate that FcγR binding is also crucial for antitumor efficacy of TIGIT inhibition in MM. Together, these data position TIGIT inhibition after ASCT as a logical immunotherapeutic approach to target the DNAM-1^+^ Teff cells described here in the donor grafts of patients with MM.

We have previously demonstrated that memory T cells in myeloma-experienced donor grafts are key mediators of antimyeloma activity in mice undergoing ASCT (1). Therefore, targeting myeloma-reactive T cells in donor grafts could deepen responses after ASCT and further prolong PFS. Our data suggests that T cells that reside in the BM in MM are likely to contain effectors with the most myeloma-specific immunity. The impact of stem cell mobilization on these cells remains unclear. Nevertheless, the frequency of DNAM-1^+^CD28^+^TIGIT^+^ T cells, the optimal target population for TIGIT inhibition, was not affected by G-CSF–mediated stem cell mobilization in our clinical cohorts. Furthermore, although G-CSF is known to alter cytokine production in T cells, the effect on IFN-γ and TNF-α secretion, key antitumor cytokines, is minimal (34). We did note transient changes in the frequency of T cell subsets, Tn and Tem, between pre– and post–G-CSF PBMC samples, which were not sustained in the apheresis product. These effects are likely due to the induction of protease activity following myeloid expansion by G-CSF, which results in transient cleavage of some surface proteins (35). Identifying the CD8^+^ T cell phenotypes in PBSC grafts that are associated with long-term PFS after ASCT in patients is another pertinent clinical objective, which requires analysis of large, well-defined clinical cohorts. What is known is that intervention with ASCT, typically as consolidation after initial induction therapy, is associated with superior PFS, although not overall survival (36). We and others have also demonstrated that administration of immunotherapy early in the disease course generates superior tumor control and that some immunotherapies have better efficacy when combined with SCT compared with nontransplant settings (1, 2, 37). Nonetheless, despite early intervention and combination with SCT, the response to αTIGIT was bimodal in our preclinical model. By 8 weeks after SCT, mice either had controlled myeloma or had uncontrolled relapse that was ultimately fatal. These bimodal responses to immunotherapies are seen with other targets such as PD-1 inhibition and CD137 agonism (1, 2, 37). Resistance to immunotherapy, and cellular therapies, is driven by many factors, including accumulation of T cells that have lost their ability to self-renew (38, 39), an immunosuppressive microenvironment (40), loss of T cell function (2, 41–43), and tumor-intrinsic resistance mechanisms (44, 45).

Several immunotherapy resistance mechanisms can be overcome by combining drugs with nonoverlapping mechanisms of action. In our preclinical model T cell clonality was increased within effector and exhausted CD8^+^ T cell clusters in the BM and the frequency of cytotoxic effector cells was increased in αTIGIT-treated mice. Additionally, the phenotype of memory T cells, including increased expression of the gene encoding IL-7R, was altered by lenalidomide exposure. The combination of both therapies significantly enhanced effector function in CD8^+^ T cells and increased the frequency of memory T cells in the BM. These are clinically relevant phenotypes, as clonal expansion of cytotoxic T cells has been associated with exceptional control of MM in patients and loss of memory T cells has been associated with progression from monoclonal gammopathy of undetermined significance (MGUS) to active myeloma (46, 47). Additionally, the combination of αTIGIT with lenalidomide increased *CXCR6* gene expression in cytotoxic T cells, which putatively positions these cells to interact with dendritic cells in the tumor microenvironment that provides critical survival signals (48). The combination also drove a unique T cell phenotype within the Tex cell clusters that was associated with high expression of *Fcerg1*, a gene associated with an innate-like T cell phenotype that is highly cytotoxic (25). Interestingly, the myeloid compartment and NK cell numbers were unaffected by the combination, while CD8^+^ T cell depletion completely abrogated protection from relapse. Thus, the synergistic activity of this combination approach is likely mediated by driving differential phenotypes and targeting different resistance mechanisms within the same immune cell subset, CD8^+^ T cells, rather than by targeting the tumor microenvironment and CD8^+^ T cells, for example. Nonetheless, we observed a bimodal response to the combination approach whereby some mice were still resistant to immunotherapy. The aforementioned immune mechanisms that underpin resistance to monotherapy likely contribute to resistance here as well. We speculate that inadequate infiltration of tumor-specific Teff cells into myeloma lesions may play an additional role in facilitating tumor outgrowth. Notably, our studies have not addressed potential additional synergy mediated by direct antimyeloma effects of lenalidomide, as our Vk*MYC cells are on a wild-type background (49).

In conclusion, TIGIT, but not PD-1, was expressed on activated CD8^+^ T cells in the PBSC grafts of patients with MM and our preclinical data highlight that the antimyeloma efficacy of TIGIT inhibition was highly dependent on FcγR engagement. Furthermore, the combination of TIGIT inhibition with IMiDs provided synergistic myeloma control, presenting a logical approach to augment PFS after transplantation. Indeed, a clinical trial combining an Fc-enabled αTIGIT antibody (EOS884448) with a next-generation IMiD (iberdomide) is now recruiting patients with relapsed/refractory myeloma (ClinicalTrials.gov NCT05289492).

## Methods

### Human samples.

Leftover satellite vials from mobilized PBSC grafts from patients undergoing ASCT for MM or healthy donors were analyzed from the Fred Hutchinson Cancer Center (Seattle). A healthy donor cohort from the QIMR Berghofer Medical Research Institute (Brisbane) was used as a comparator. Healthy volunteer PB samples were collected and PBMCs isolated using Ficoll-Paque PLUS (Cytiva) prior to cryopreservation in IMDM (GIBCO by Life Technologies) media containing 20% FBS (GIBCO by Life Technologies) and 10% DMSO. Healthy sibling transplant donors were administered G-CSF for 4 consecutive days and PB collected before and after G-CSF mobilization, together with a sample of the apheresis product. Spherotech Rainbow Calibration Particles (8 peak) from the same lot number were employed to calibrate the voltages across BD FACSymphony instruments used to acquire human data from the Seattle and Brisbane cohorts (cohorts 1 and 2, respectively).

### Mice and BM transplantation.

C57BL/6J and CRBN mice were purchased from The Jackson Laboratory. CRBN-transgenic mice are genetically engineered to allow engagement of thalidomide and its derivatives (including lenalidomide) with a modified murine CRBN (cereblon) resulting in degradation of the IMiD targets, Aiolos and Ikaros (50). Mice were housed in sterile microisolator cages and received acidified (pH 2.5), autoclaved water and normal chow. Experiments were performed with female mice at 8–12 weeks of age. Recipients were monitored daily and sacrificed when hind limb paralysis occurred, or clinical scores reached 6 or higher (20). Recipient mice were injected with Vk12653 (51) 2 weeks prior to BM transplantation (1 × 10^6^; MM-bearing) and were transplanted as previously described with cell doses detailed in the figure legends (2, 52). M-band was quantified in serum as previously described (1, 51). Mice were treated with 100 μg Fc-live αTIGIT (mIgG2a, EOS-448 surrogate, iTeos Therapeutics), 100 μg Fc-dead αTIGIT (mIgG2a, EOS-448 with N297A mutation surrogate, iTeos Therapeutics), or mIgG2a via i.p. injection twice weekly from day 0 to day +35 after SCT (19). Lenalidomide (50 mg/kg; Sigma-Aldrich) or vehicle was administered via daily oral gavage for 21 days from day +14 after SCT. For CD8^+^ T cell depletion experiments, mice were treated with 150 μg αCD8β (clone 53.5.8, grown in-house) once a week from day 0 until 5 weeks after SCT, as previously described (1).

### Cell preparation and flow cytometry.

For BM aspirates, mice were anesthetized and 30 μL of PBS was injected into the femur and 10 μL of marrow was aspirated. For endpoint analysis, recipient mice were sacrificed 6 weeks after SCT and cells from BM and blood were harvested. Mouse blood samples underwent red blood cell lysis prior to surface staining. For analysis of human PBSC samples, frozen satellite vials and healthy PBMC samples were thawed and 5 × 10^6^ total cells were stained for surface markers prior to fixation for intracellular staining. For all flow cytometry processing, isolated cells were incubated with Fc-block (2.4G2 for mouse, TruStain FcX for human) prior to staining for 30 minutes on ice with antibodies listed in Supplemental Table 3. For intracellular staining, cells were fixed and permeabilized with eBiosciences Foxp3 Transcription Factor Staining Buffer Kit prior to intracellular staining at room temperature for 60 minutes. To measure cytokine production, mouse cells were stimulated for 4 hours at 37°C with PMA (500 ng/mL) and ionomycin (50 ng/mL) (Sigma-Aldrich) with Brefeldin A (BioLegend). All samples were acquired on a FACSymphony A3 (BD Biosciences) and analyzed using FlowJo (v10). FlowSOM, which used a self-organizing map method of data visualization, analysis was performed on concatenated samples, 4,000 CD8^+^ T cells per mouse from each independent experiment or 5,000 CD8^+^ T cells per patient sample (12). Descriptions of markers expressed in each population are included in Supplemental Tables 1 and 2 for mouse experiments. Populations with similar, overarching phenotypes were grouped according to Supplemental Tables 1 and 2 and the two experiments were pooled to allow quantification of each broad phenotype across treatment groups. Lists of human and mouse antibodies used for these experiments are included in Supplemental Table 3.

### Single-cell RNA and TCR sequencing.

Mice were transplanted and treated with Fc-live αTIGIT and lenalidomide as described above. BM was harvested 4 weeks after SCT and CD8^+^ T cells were sort purified (>98% purity) from 5 mice pooled per group. Samples were captured with a 10× Genomics Chromium Controller and libraries were generated using the 10× Genomics Chromium Next GEM Single Cell 5′ v2 (Dual Index) kit according to the manufacturer’s instructions. All libraries were sequenced, with a target of 20,000 reads per cell for gene expression and 5,000 reads per cell for TCR libraries, on a NextSeq 2000 in 1 run (Illumina P3 kit). Illumina BCL files were demultiplexed and processed using cellranger (https://support.10xgenomics.com/single-cell-gene-expression/software/downloads/latest). To achieve similar read depth of gene expression libraries across samples, the cellranger aggregate function was used to downsample reads, yielding an expression matrix of gene counts per cell. Cells meeting the following thresholds were kept for downstream analysis: unique molecular indices per cell greater than 2,500 and less than 25,000, percentage mitochondrial RNA less than 15%, and cells that exhibited expression of *Cd3e*/*g* and *Cd8a* genes. Dimensionality reduction on this matrix was performed using Monocle3 (53–55) with standard parameters using the top 2,000 variable features. Cell subsets were identified by Leiden clustering (resolution 2 × 10^–4^) and using FindAllMarkers() (56–58). For TCR libraries, the cellranger vdj function was used to reconstruct single-cell TCR sequences. TCR data were analyzed using scRepertoire (59). The TCR clonality for each gene expression cluster was evaluated using Simpson’s Clonality Index, which ranges from 0 (all clonotypes of size 1) to 1 (a single expanded clonotype).

### Statistics.

Data presented as mean ± SEM and a *P* value of less than 0.05 was considered significant. Survival curves were generated using Kaplan-Meier estimates and treatment groups were compared by log-rank (Mantel-Cox) test; the Benjamini-Hochberg procedure was performed to correct for multiple comparisons. Statistical tests are described in the figure legends and M-bands were modeled as previously described (1, 2).

### Data availability.

The RNA sequencing data generated in this study have been deposited in the NCBI Gene Expression Omnibus database (GEO GSE220195) and all code used for bioinformatic analyses is available at https://github.com/furlan-lab/tigit_inhibition

### Study approval.

The use of human samples for research was approved by the Fred Hutchinson Cancer Research Center IRB and all human participants provided written informed consent. In the Brisbane samples, ethics approval was obtained from the Human Research Ethics committees of QIMR Berghofer and The Royal Brisbane and Womens’ Hospital, with written informed consent obtained from all participants. All animal procedures were performed in accordance with protocols approved by Fred Hutchinson Cancer Research Center IACUC.

## Author contributions

SAM designed and performed experiments, analyzed data, and wrote the manuscript. OGW, KSE, SDO, ADC, DPS, CRS, SRWL, ST, NSN, TS, SNF, and AV performed experiments and/or data analyses. GD provided crucial reagents and informed experimental design. PZ, MK, AS, and LAH helped with experimental design and/or sample acquisition. GRH conceived and supervised the study and helped write the manuscript. All authors edited and approved the final manuscript.

## Supplementary Material

Supplemental data

## Version 1 12/13/2022

In-Press Preview

## Version 2 02/15/2023

Electronic publication
