# Supplementary material for: TIGIT inhibition and lenalidomide synergistically promote antimyeloma immune responses after stem cell transplantation in mice
Source: J Clin Invest. 2023 Feb 15;133(4):e157907. doi: 10.1172/JCI157907 (PMC9927935; doi:10.1172/JCI157907)
Supplement: Supplemental data [file jci-133-157907-s007.pdf]

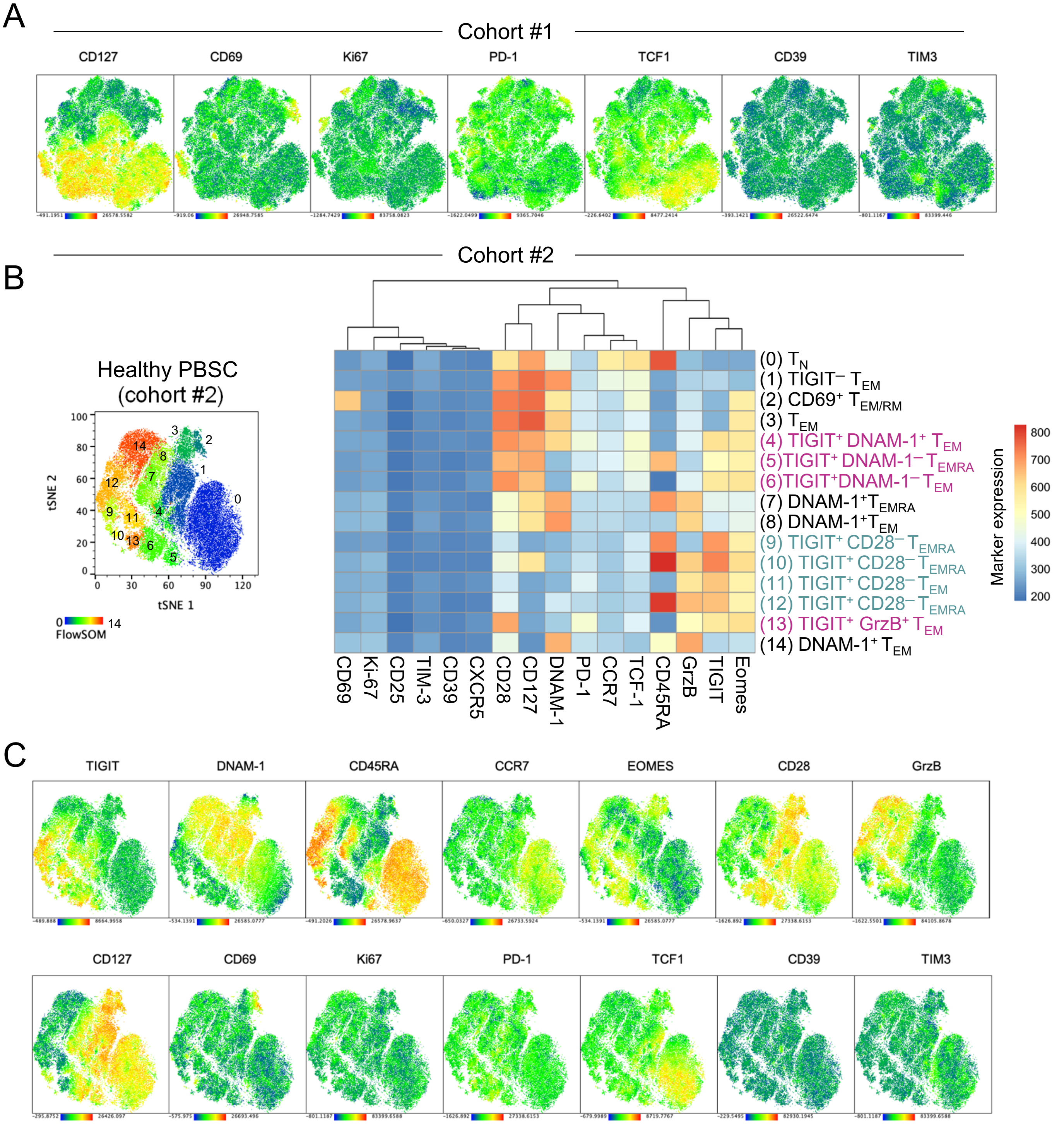

Supplementary Figure 1: CD8 T cell clustering in healthy peripheral blood stem cell grafts. Mobilized peripheral blood stem cell (PBSC) grafts from patients undergoing ASCT for myeloma (MM) were thawed and stained for analysis via flow cytometry alongside healthy PBSC grafts (total *n* = 14 myeloma; *n* = 15 for Healthy PBSC). **(A)** TSNE plots from all myeloma samples and three healthy controls colored by expression of markers of interest in cohort 1. **(B)** TSNE plot of CD8 T cells, colored by FlowSOM populations, in healthy PBSC grafts in cohort 2 (from Brisbane; *n* = 12) and heatmap of maker expression (MFI) across FlowSOM CD8 T cells populations. TIGIT$^+$ populations are colored blue (CD28$^-$) or purple (CD28$^+$) to indicate putative senescence vs activation respectively. **(C)** TSNE plots from samples in (B) colored by expression of markers of interest.

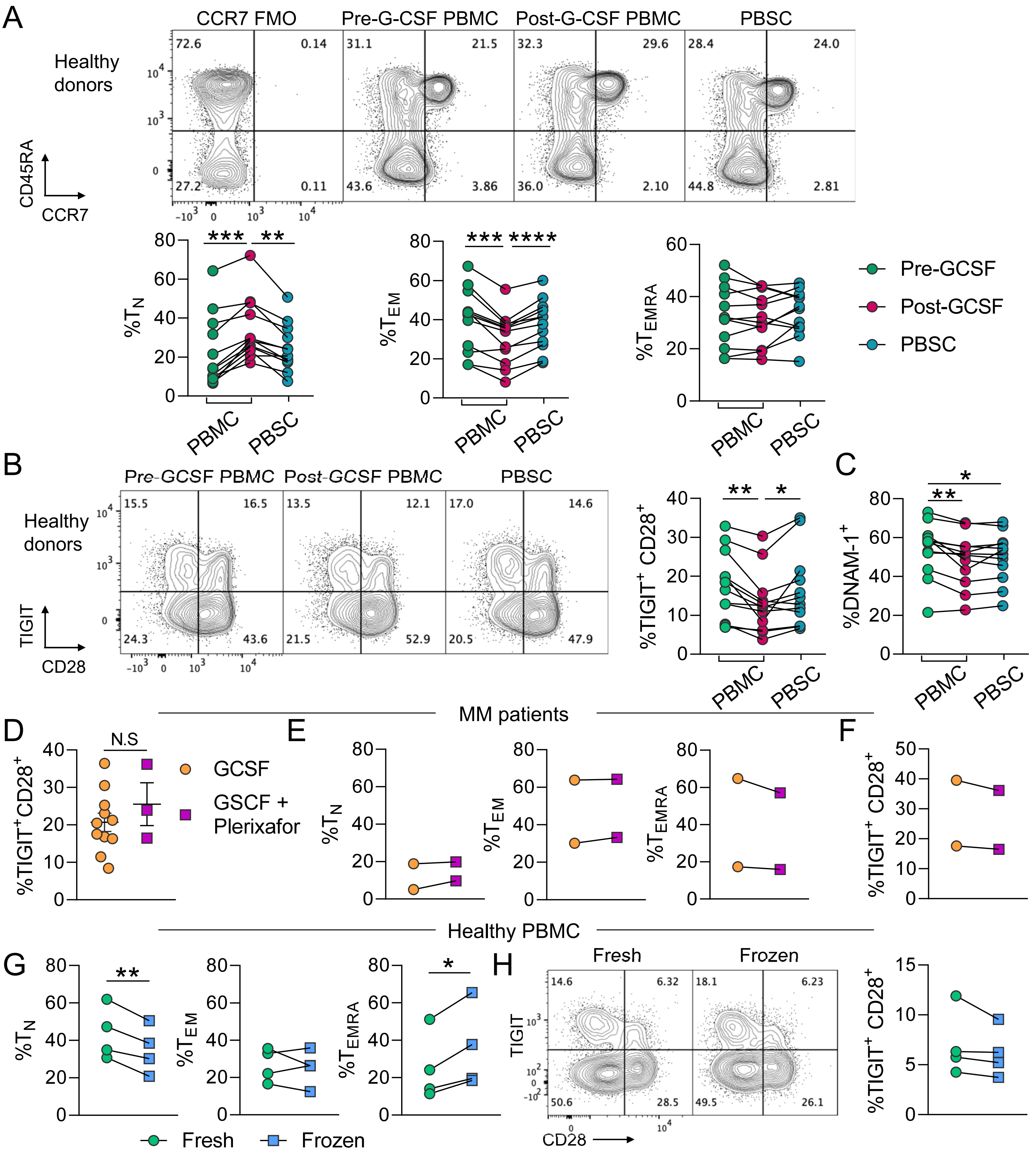

Supplementary Figure 2: CD8 T cell clustering in healthy peripheral blood stem cell grafts. (A-C) Peripheral blood mononuclear cells (PMBC) from healthy patients before and after G-CSF mobilization and peripheral blood stem cell grafts (PBSC) were thawed and CD8 T cells were analyzed using flow cytometry (FACS) (*n* = 12). **(A)** Representative FACS plots of CCR7 and CD45RA expression and frequencies of T cell subsets. **(B)** FACS plots of TIGIT and CD28 expression with frequency of TIGIT$^+$CD28$^+$ cells within CD8 T cells. **(C)** Frequency of DNAM-1$^+$ cells within TIGIT$^+$ CD28$^+$ T cells. **(D-F)** PBSC grafts from patients undergoing ASCT for myeloma (MM) were thawed for FACS analysis (*n* = 14). **(D)** Frequency of TIGIT$^+$CD28$^+$ CD8 T cells in patients mobilized with G-CSF alone (*n* = 11) or G-CSF with plerixafor (*n* = 3). **(E)** Frequency of CD8 T cell subsets and **(F)** TIGIT$^+$CD28$^+$ T cells in patients mobilized with G-CSF alone followed by G-CSF with plerixafor on a subsequent day. **(G-H)** PBMCs from healthy volunteers were freshly isolated for FACS analysis before and after cryopreservation (*n* = 4). **(G)** Frequency of CD8 T cell subsets. **(H)** Representative FACS plots and frequency of TIGIT$^+$CD28$^+$ CD8 T cells. RM one-way ANOVA with Tukey's test or paired t test. *p<0.05, **p<0.01, ***$p<0.001$. ****$p<0.0001$.

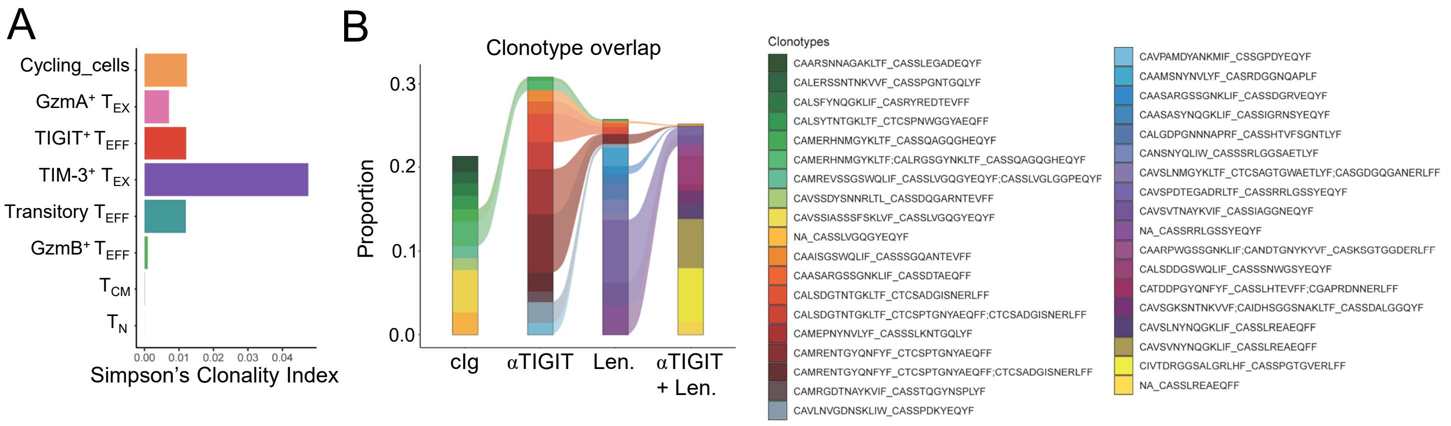

Supplementary Figure 3: T cells are clonally expanded in T_{EX} and T_{EFF} clusters with clonotype overlap between mice treated with αTIGIT and lenalidomide monotherapy and combination therapy. CRBN or B6 recipients were transplanted with 10 x 10^6 BM with 2 x 10^6 T cells from CRBN or B6 donors and then treated with 100 µg of αTIGIT or isotype control (cIg) twice a week from D0 and daily lenalidomide (50 mg/kg; Len.) or vehicle from D+14 until 4 weeks post-SCT. Mice were sacrificed at week 4 and CD8 T cells were sorted for 5' single cell RNA sequencing (*n* = 5/group). **(A)** Simpson's Clonality Index within clusters. **(B)** Clonotype overlap across treatment groups. Lines between groups depict clonotype overlap between treatment groups and colors indicate individual TCR clones. This is the same graph presented in Figure 3G, included here with the legend describing specific TCR clonotypes.

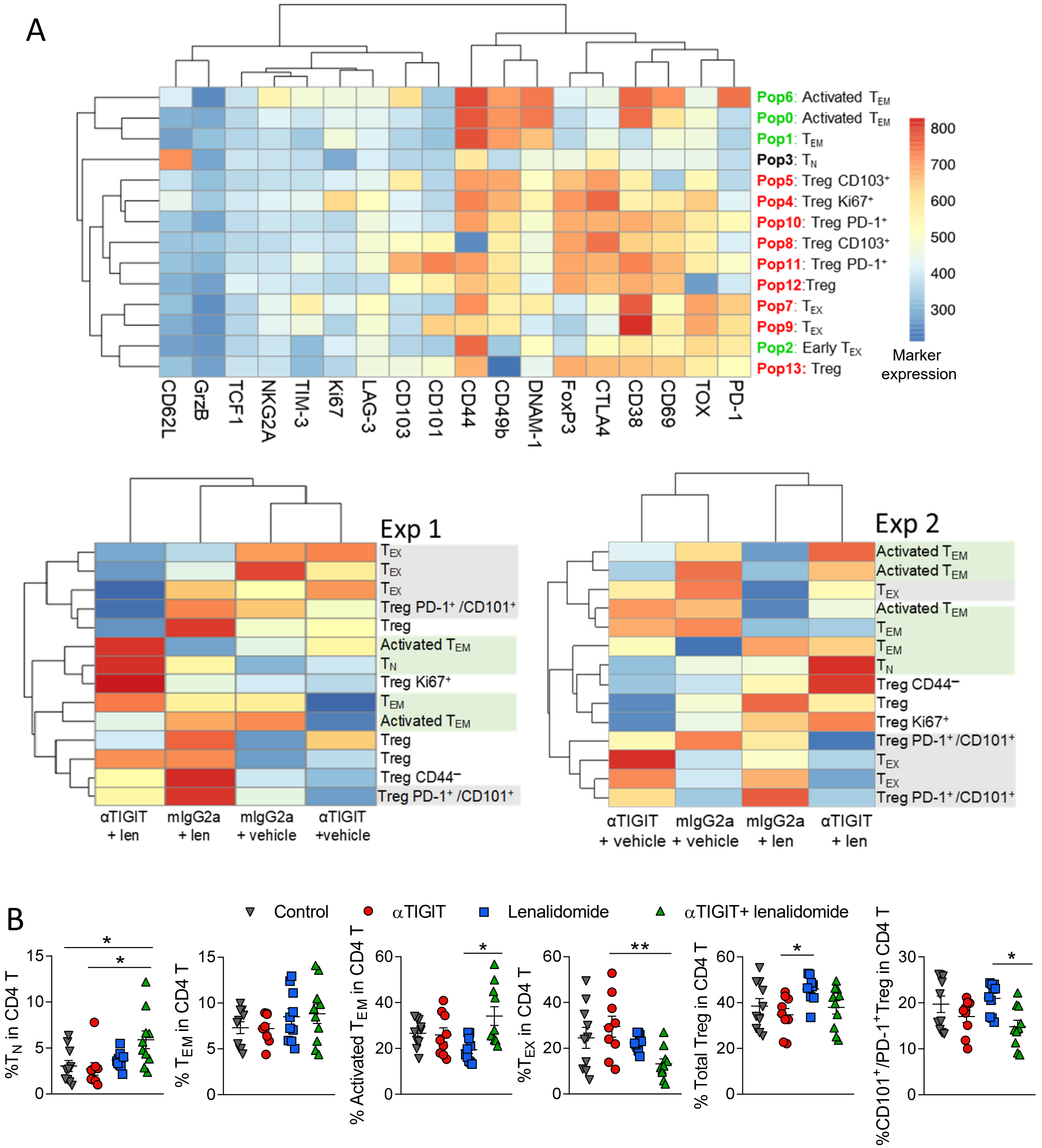

Supplementary Figure 4: The combination of αTIGIT and lenalidomide has modest effects on CD4 T cells in the BM. CRBN or B6 recipients were transplanted with 10 x 10^6 BM with 2 x 10^6 T cells from CRBN or B6 donors and then treated with 100 µg of αTIGIT or cIg twice a week from D0 and daily lenalidomide (50 mg/kg) or vehicle from D+14 until 5 weeks post-SCT. Mice were sacrificed at week 6 and BM and blood were harvested for analysis by flow cytometry (n = 10/group from 2 independent experiments). **(A)** Representative heatmap of marker expression (MFI) in each population of CD4 T cells identified using FlowSOM (top) and heatmaps of the relative mean frequency of each population across treatment groups from two replicative experiments (bottom). T_N = CD62L$^+$ CD44$^-$, T_{CM} = CD62L$^+$ CD44$^+$, T_{EM} = CD62L$^-$ CD44$^+$, Treg = FoxP3$^+$ **(B)** Quantification of broader phenotypes (inc. one or more populations identified by FlowSOM) across treatment groups. Descriptions of individual populations and how they are grouped is included in Supplementary Table 2. Data represent mean ± SEM. One-way ANOVA with Tukey's test or Kruskal-Wallis test with Dunn's multiple comparisons test. *p<0.05, **p<0.01.

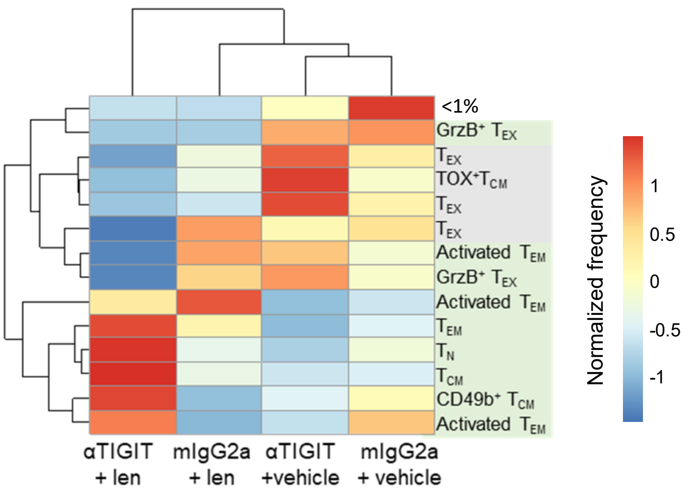

Supplementary Figure 5: Relative frequency of FlowSOM-generated populations in CD8 T cells across treatment groups in a replicative experiment. CRBN or B6 recipients were transplanted with 10 x 10^6 BM with 2 x 10^6 T cells from CRBN or B6 donors and then treated with 100 µg of αTIGIT or cIg twice a week from D0 and daily lenalidomide (50 mg/kg) or vehicle from D+14 until 5 weeks post-SCT. Mice were sacrificed at week 6 and BM and blood were harvested for analysis by flow cytometry. Heatmap of the relative mean frequency of each population across treatment groups. T_N = CD62L$^+$ CD44$^–$, T_{CM} = CD62L$^+$ CD44$^+$, T_{EM} = CD62L$^–$ CD44$^+$

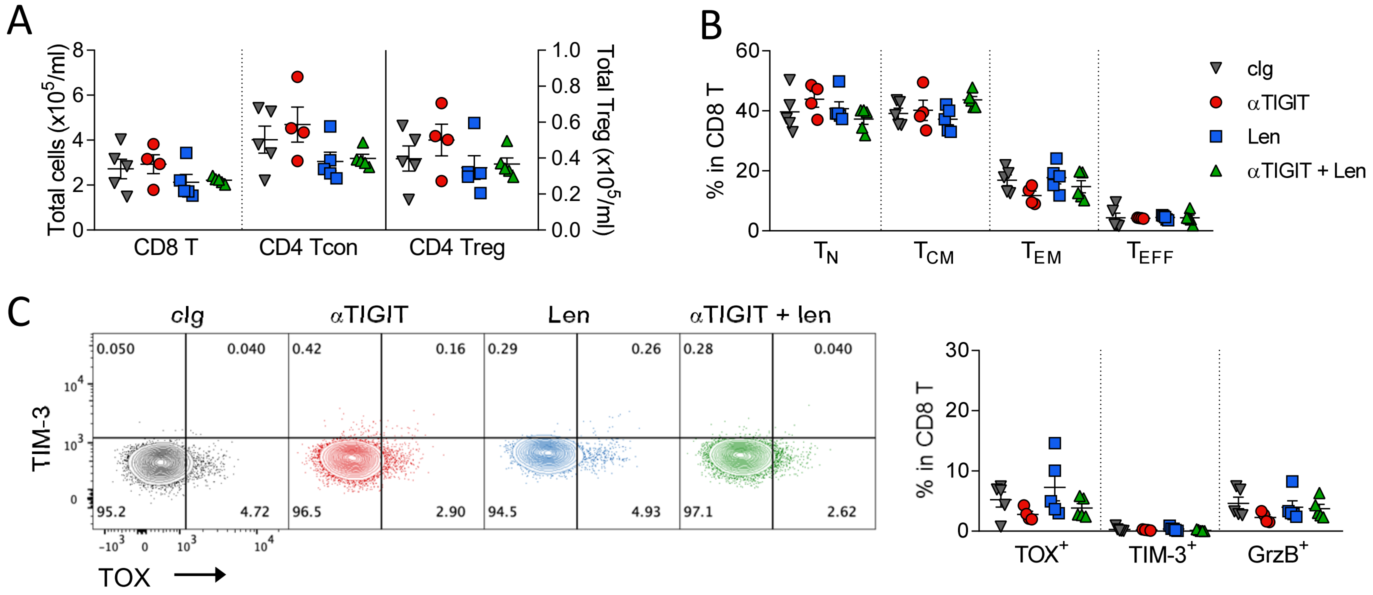

Supplementary Figure 6: Immunological effects of αTIGIT and lenalidomide are bone marrow specific. CRBN or B6 recipients were transplanted as described in Figure 2 and blood was collected at week 6 post-SCT. **(A)** Quantification of T cell subsets in peripheral blood. **(B)** CD8 T cell differentiation in blood (as described in Figure 5 and T_{EFF} = CD62L$^-$ CD44$^-$). **(C)** Representative flow cytometry plots of TIM-3 and TOX expression in CD8 T cells from peripheral blood and quantification of TOX, TIM-3 and granzyme B (GrzB) expression. n = 5/group from 1 experiment. One-way ANOVA with Tukey's test. Data represent mean ± SEM.

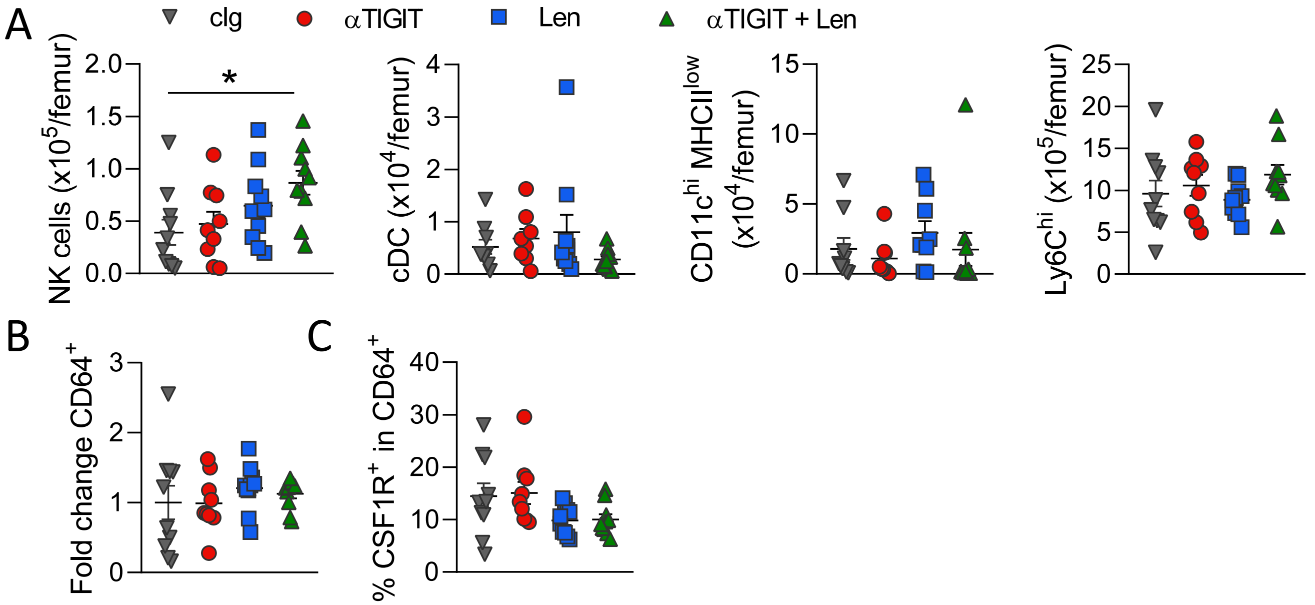

Supplementary Figure 7: Natural killer and myeloid cells are unaffected by the combination of lenalidomide and TIGIT. CRBN or B6 recipients were transplanted with 10 x 10^6 BM with 2 x 10^6 T cells from CRBN or B6 donors and then treated with 100 μg of αTIGIT or cIg twice a week from D0 and daily lenalidomide (50 mg/kg) or vehicle from D+14 until 5 weeks post-SCT. Mice were sacrificed at week 6 and BM was harvested for analysis by flow cytometry (n = 10/group from 2 experiments). **(A)** Total number of natural killer (NK) cells, conventional dendritic cells (cDC), $MHCII^{low}$ DCs, and $Ly6C^{hi}$ monocytes per femur. **(B)** Fold change in total number of $CD64^+$ macrophages and **(C)** the frequency of CSF1R expression on macrophages. Data represent mean ± SEM. One-way ANOVA with Tukey's test. *p<0.05

Supplemental Tables:

Supplementary Table 1: Expression of flow cytometry markers within each population of mouse CD8 T cells across two independent experiments.

**Description**	**Experiment 1**	**Experiment 2**
CD49b^{+} T_{RM}	**Pop 0:** DNAM-1+ CD62L+ NKG2A+ CD69+ CD49b+ CD38+ CD44+	**Pop 2**: DNAM-1+ CD62L+ NKG2A+ CD69+ CD49B+ CD38+ CD44+
Naïve T	**Pop 13**: CD62L+ DNAM-1+	**Pop 0**: CD62L+ DNAM-1+
T_{CM}	**Pop 10**: DNAM-1+ CD62L+ CD44+	**Pop 1**: DNAM-1+ CD62L+ CD44+
T_{EX}	**Pop 5**: TOX+ PD-1+ CD101+ CD38+ CD44+ TIM3+ LAG3+	**Pop 11**: TOX+ PD-1+ CD101+ CD38+ CD44+ TIM-3+ LAG3+ Ki67+
	**Pop 3**: TOX+ PD-1+ CD101+ CD38+ CD44+ CD49b+ DNAM-1+	**Pop 13**: TOX+ PD-1+ CD101+ CD38+ CD44+
	**Pop 9**: TOX+ PD-1+ CD38+ CD44+	**Pop 9**: TOX+ PD-1+ CD38+ CD44+ LAG3+
	**Pop 6 + 4**: TOX+ PD-1+ CD101+ CD38+ CD44+TIM3+ LAG3+ CD49b+	
GrzB^{+} $T_{EM/EX}$	**Pop 2**: GrzB+ DNAM-1+ Ki67+ CD38+ CD44+ TIM3+ TOX+ PD-1+	**Pop 10**: GrzB+ TIM-3+ TOX+ PD-1+ CD38+ CD44+ LAG3+
		**Pop 8**: GrzB+ DNAM-1+ Ki67+ CD38+ CD44+ CD49b+
T_{EM}	**Pop 12**: DNAM-1+ CD44+	**Pop 5**: DNAM-1+ CD38^{low} CD44+
Activated T_{EM}	**Pop 1**: DNAM-1+ CD38+ CD44+ CD49b+	**Pop 12**: DNAM-1+ CD38+ CD44+ PD-1+
	**Pop 8**: CD69+ CD38+ CD44+ DNAM-1-	**Pop 4**: DNAM-1+ CD69+ CD38+ CD44+ CD49B+
	**Pop 7**: DNAM-1+ NKG2A+ PD-1+ CD69+ CD49b+ CD38+ CD44+	**Pop 6**: CD38+ CD44+ PD-1+
Tox^{+} T_{CM}		**Pop 7**: CD62L+ LY108+ CD38+ CD44+ PD-1+ TOX+

Supplementary Table 2: Expression of flow cytometry markers within each population of mouse CD4 T cells across two independent experiments.

Description	Experiment 1	Experiment 2
Naïve T	**Pop 3**: CD62L+	**Pop 4**: CD62L+
T_{EX}	**Pop 7**: TOX+ PD-1+ TIM-3+ LAG3+ CD44+ DNAM-1+ CD38+	**Pop 13**: TOX+ PD-1+ LAG3+ CD44+ DNAM-1+ CD38+ Ly108+
	**Pop 9**: TOX+ PD-1+ CD44+ CD101+ CD38+	**Pop 6:** TOX+ PD-1+ CD44+ CD38+
	**Pop 2**: PD-1+ TOX+ CD44+ DNAM-1+	**Pop 9:** TOX+ PD-1+ CD101+ CD38+
Treg: CD44 neg	**Pop 8:** FoxP3+ CTLA4+ CD44-	**Pop 5:** FoxP3+ CTLA4+ CD62L+ CD44-
Treg: Ki67+	**Pop 4:** FoxP3+ CTLA4+ TOX+ CD44+ Ki67+ LAG3+	**Pop 8:** FoxP3+ CTLA4 high LAG3+ Ki67+ CD44+ CD62L+
Treg: PD-1+/CD101+	**Pop 10:** FoxP3+ CTLA4+ CD69+ PD-1+ CD44+ CD38+	**Pop 12:** FoxP3+ CTLA4+ CD69+ LAG3+ PD-1+ TOX+ CD44+
	**Pop 11:** FoxP3+ CTLA4+ CD69+ CD101+ PD-1+ CD38+ CD44+	**Pop 10:** FoxP3+ CTLA4+ CD69+ CD44+ CD101+
Treg	**Pop 5:** FoxP3+ CTLA4+ CD44+	**Pop 7:** FoxP3+ CTLA4+CD44+
	**Pop 12 + 13:** FoxP3+ CTLA4+ CD69+ CD38+ CD44+	
T_{EM}		**Pop 3:** CD44+ Ly108+
	**Pop 1:** DNAM-1+ CD49b+ CD44+	**Pop 11:** CD44+ CD101+ CD49b+
Activated T_{EM}	**Pop 6:** PD-1+ CD69+ CD38+ DNAM-1+ CD49b+ CD44+	**Pop 0:** CD69+ CD38+ DNAM-1+ CD49b+ CD44+
	**Pop 0:** CD38+ DNAM-1+ CD49b+ CD44+ CD103+ NKG2A+	**Pop 1:** CD38+ DNAM-1+ CD49b+ CD44+ CD69+ NKG2A+
		**Pop 2:** CD38+ DNAM-1+ CD44+ CD49b+ Ki67+ LAG3+

Supplementary Table 3: Flow cytometry antibodies

Marker	Clone	Fluorochrome	Company
Mouse:			
CD226	TX42.1	BV650	Biolegend
CD101	Moushi101	AF700	eBioscience
CD69	H1.2F3	BV786	Biolegend
CD62L	MEL-14	AF700<br>BV480	Biolegend<br>BD Bioscience
CD4	GK1.5	BUV496	BD Bioscience
CD3	145-2C11	BV711	Biolegend
CD38	T10	PE-Cy7	Biolegend
CD8	53-6.7	APC-Cy7<br>BUV805	Biolegend<br>BD Bioscience
PD-1	29F.1A12<br>RMP1-30<br>J43	BV421<br>PE-Cy7<br>BUV737	Biolegend<br>Biolegend<br>BD Bioscience
CD44	IM7	BV421, APC-Cy7	Biolegend
CD90.2	53-2.1	BV605	Biolegend
TIM-3	RMT3-23	FITC<br>BV605	eBioscience<br>Biolegend
TIGIT	1G9	BV421	BD Bioscience
NKp46	29A1.4	PE	Biolegend
Ly108	13G3	BUV661	BD Bioscience
TOX	TXRX10	eFluor660	eBioscience
FoxP3	FJK-16s	PE-Cy5	eBioscience
NRP-1	3E12	PerCp/Cy5.5	Biolegend
CD49b	HMα2	BUV563	BD Bioscience
CD103	2E7	BUV661	BD Bioscience
Granzyme B	QA16A02	PE-Dazzle594	Biolegend
Perforin	S16009A	PE	Biolegend
CD122	TM-β1	BB700	BD Bioscience
CD45	30-F11	BUB395	BD Bioscience
NKG2A	20d5	BV605	Biolegend
Human:			
CD3	SK7	BUV395	BD Bioscience
CD4	SK3	BUV805	BD Bioscience
CD8	RPA-T8	BUV496	BD Bioscience
CD127	A019D5	PE-Cy5	Biolegend
CD25	2A3	BV605	BD Bioscience
PD-1	EH12.2H7	BV786	Biolegend
TIGIT	A15153G	BV421	Biolegend
CD69	FN50	BUV563	BD Bioscience
CD28	CD28.2	BUV737	BD Bioscience
Granzyme B	GB11	BV510	BD Bioscience
Ki67	B56	BV650	BD Bioscience
CD39	TU66	BB515	BD Bioscience
TIM-3	7D3	BB700	BD Bioscience
TCF-7/TCF-1	S33-966	PE	BD Bioscience
CD45RA	HI100	APC-Cy7	BioLegend

CCR7	2-L1-A	BUV661	BD Bioscience
EOMES	WD1928	PE-Dazzle594	ThermoFischer
CXCR5	RF8B2	BV750	BD Bioscience
DNAM-1	DX-11	BV711	BD Bioscience
